# A reliable facility location design model with site-dependent disruption in the imperfect information context

**DOI:** 10.1371/journal.pone.0177104

**Published:** 2017-05-09

**Authors:** Lifen Yun, Xifu Wang, Hongqiang Fan, Xiaopeng Li

**Affiliations:** 1 School of Traffic and Transportation, Beijing Jiaotong University, Beijing, China; 2 School of Modern Post, Beijing University of Posts and Telecommunications, Beijing, China; 3 Department of Civil and Environmental Engineering, University of South Florida, Tampa, United States of America; Beihang University, CHINA

## Abstract

This paper proposes a reliable facility location design model under imperfect information with site-dependent disruptions; i.e., each facility is subject to a unique disruption probability that varies across the space. In the imperfect information contexts, customers adopt a realistic “trial-and-error” strategy to visit facilities; i.e., they visit a number of pre-assigned facilities sequentially until they arrive at the first operational facility or give up looking for the service. This proposed model aims to balance initial facility investment and expected long-term operational cost by finding the optimal facility locations. A nonlinear integer programming model is proposed to describe this problem. We apply a linearization technique to reduce the difficulty of solving the proposed model. A number of problem instances are studied to illustrate the performance of the proposed model. The results indicate that our proposed model can reveal a number of interesting insights into the facility location design with site-dependent disruptions, including the benefit of backup facilities and system robustness against variation of the loss-of-service penalty.

## Introduction

In the early studies on the facility location design problem, the facilities, once built, will remain functioning all the time. Based on this assumption, scientists propose a number of traditional models for the different facility location design problems (see Drezne [1] and Daskin [2] for a review on this topic). These works help decision makers obtain economic facility location design assuming all facilities are operating normally all the time. However, in recent year, there are increasing recognitions on the fact that constructed facilities may be disrupted any time during operations by either anthropogenic or natural disastrous events, such as the 2002 west-coast port lockout [3], the 2003 massive power outage [4] and 2012 Hurricane sandy [5]. Snyder and Daskin [6] pointed out that the traditional facility location design models that ignore facility disruption possibilities often yield suboptimal facility location design. In order to obtain the optimal facility location design considering possible facility disruptions, a number of reliable facility location models have been proposed.

In the reliable facility location design models, a huge number of facility disruption scenarios should be handled even if each facility has two states (operating or not). Each scenario is a unique combination of all facility states. In order to reduce the complexity of modeling reliable facility location problems, most existing studies assume that facility disruptions take place independently with an identical probability. However, this assumption may not reasonably reflect realistic facility reliability. In the real world, facilities likely have distinguished features (e.g., capacity, equipment, labor skills, etc.) and are subject to different levels of disruption risks depending on their geographic environment (e.g., a place closer to a river is subject to a higher flooding risk). As such, a facility is likely subject to a site-dependent disruption probability that varies across the space. Such site-dependent disruptions significantly raise the difficulty of describing and modeling relevant facility location design problems. Thus, only limited works have been done in the facility location literature to address site-dependent disruptions [7–11]. All these studies investigate problems under perfect information; i.e., customers know the exact real-time information of facility states and always visit the nearest functional facility directly in any disruption scenario.

In many real world problems, however, customers may not get the real-time information of facility states. Even in the information-rich environment, sharing real-time information remains a critical challenge due to sensor technology limitations [12] and various institutional and technological barriers [13]. Further, a severe disruption scenario is often accompanied with losses of communication infrastructures [14], which may cut off customers' communications with their services facilities and thus make them blind to facility disruption states. Under such circumstance, which we refer as "imperfect information", a customer is likely to adopt a “trial-and-error” strategy such that they keep visiting facilities according to a pre-assigned sequence regardless of the disruption scenario until they arrive at the first operating facility or give up looking for the service. This "trial-and-error" visiting behavior further complicates quantification of associated operational costs. Only a few studies have been conducted for suitable reliable facility location design [15, 16] under imperfect information. All these studies assume that all candidate facilities have the identical independent disruption probabilities, while site-dependent disruptions under imperfect information remain unaddressed probably due to modeling difficulties.

To bridge this gap, this study proposed an innovative solution approach for the reliable facility location design problem with site-dependent disruptions under imperfect information. This study overcomes the aforementioned modeling challenges and formulates a compact polynomial-sized mixed integer programming model for this problem without enumerating the exponential number of disruption scenarios. This model is further linearized to enable it to be solved with existing mixed linear integer programming solution methods. A case study is constructed to show that this compact model can be efficiently solved by off-the-shelf commercial solver and to draw insights into the impact of disruption site-dependence and other key parameters on the optimal system costs and location design. This development enables planners to design reliable infrastructure systems that not only have appealing performance in the normal scenario but also provide reasonable service when facilities are disrupted with site-dependent probabilities and customers have no way to access facility disruption states.

The remainder of this paper is organized as follow. The next section reviews the relevant literature on the facility location design problem. The third section states the research problem, formulates it as the non-linear integer programming model, and further simplifies it as an equivalent linear integer programming model that can be directly fed into a commercial solver. The fourth section conducts a case study to illustrate applications of the proposed model and draw relevant managerial insights. The last section concludes this work and briefly discusses future research directions.

## Literature review

The studies on facility location design problems that aim to obtain the optimal economic benefit for decision makers have been extended to various industries (e.g., logistics, energy system, traffic system, etc.) in the past decades. Weber [17] first initiated pioneering study on the facility location problem which objective is the minimization of transportation costs. In the later decades, the studies on facility location design have experienced a long period of development and many classic facility location design models are proposed for solving different facility location problems in ideal conditions. Then, Daskin [2] made a review on different classic facility location design models and introduced a number of key algorithms to solve these models. Later, a number of studies that focus on solving various realistic facility location problems sprang up by the modified classic models, i.e., facility locations and network topology design [18], inventory-location [19] and joint inventory-location [20], transportation-inventory network design [21], sensor placement in municipal water networks [22], Remanufacturing Network with reverse flows [23], and multiple distribution centers under fuzzy environment [24]. These studies share the same assumption that each facility, once built, will always be operational forever. With this assumption, these studies obtain the economic facility location design that yields the minimum system cost.

In the recent decades, due to frequent anthropogenic or natural disastrous events [3–5], researchers have paid more attentions to the influence of the facility disruption. Due to the facility disruption, customers cannot obtain the service from the pre-assign facility as planning. As a result, the total system cost will increase sharply and the economic facility location design obtained by classic facility location models will become suboptimal design. To address the facility disruption problems, a number of reliable facility location models are proposed by considering possible facility disruptions which have been observed in the real world. The optimal facility location design may cause some backup facilities to be constructed in case of possible facility disruption, but for a long time period the total system cost will be reduced if the infrequent facility disruptions occur. These models have to handle a huge number of facility disruption scenarios even the facility only has two states: operating and complete disrupted. To reduce the complexity of modeling these reliable facility location problems, most of reliability models assume that the facilities are independent with identical disruption probability. The initial representative study is that Snyder and Daskin [6] proposed two reliable models based on P-median problem (PMP) and uncapacitated fixed location problem (UFLP) and presented a Lagrangian relaxation algorithm to solve them. Further, a number of reliable facility location models with independent identical disruption probability are proposed to address different issues, i.e., joint inventory-location [25], reliable sensor deployment [26], emergency service network [27, 28], supply chain [29, 30]. However, modeling facility location problems with site-dependent disruption probability are inevitable. Cui et al. [7] proposed two distinct models to study the reliable uncapacitated fixed charge location problem (RUFL) with site-dependent failure probabilities. The two models can deal with different scale problems and obtain optimal or near-optimal facility location design. Later, a number of studies extend the general facility location problem to various aspects including sensor deployment [8], biofuel supply chain [9, 10] and joint location-inventory problem [11]. The studies on the facility location problem with either independent identical or site-dependent disruption probability have the same assumption that customers have perfect information of facility states so that they can visit the nearest operating facility directly.

On the other hand, a handful of studies investigated reliable location problems under imperfect information. Berman et.al [15] proposed the reliable p-median facility location model with imperfect information by assuming that the customer always visits the closest facility one by one in any facility disruption scenario. This assumption may yield a significantly higher cost compared to the true optimal sequence. Later, Yun et al. [16] proposed another reliability model to study the RUFL problem, which assumes that the customer’s visit sequence yields the minimum transportation cost, and developed a special LR algorithm to solve it.

From the previous literature review, we find that there is no study on the reliable facility location design problem with site-dependent disruption probabilities and imperfect information. This research gap will be addressed in the following sections through developing a new reliable location model.

## Model formulation

### Problem statement

This section models the reliable facility location problem with site-dependent disruptions and imperfect information. In this problem, a number of candidate facility locations are provided for the decision maker to select. Building a facility in the candidate location needs a certain construction cost. To capture site-dependence of disruptions, we allow facilities at different locations to have different special disruption probabilities. Once the facilities are built, each customer will be assigned a number of facilities with different priorities to obtain the service. The aim of this assignment is to optimize the customers' visiting sequences. When any facility disruption scenario occurs, a customer cannot get the disruption information of her pre-assigned facilities. Thus she has to visit the pre-assigned facilities sequentially according to the assigned priorities, until she arrives at the first operating facility to obtain the service. Or she may have to give up the service when all the pre-assigned facilities have been found failed or a further trial is too expensive. Visiting each pre-assigned facility incurs a certain transportation cost to the customer. The customer bears a certain penalty cost when she gives up the service. The objective of this reliable facility location problem is to determine the optimal facility location scheme and the corresponding facility assignments for all customers in order to minimize the total expected system cost considering all possible facility disruption scenarios, including facility construction cost, customer transportation and penalty costs.

### Cost component expression

This research considers a set of spatially distributed customers and a set of facilities which provide certain service to the nearby customers in the imperfect information context. In this section, we calculate the total system cost that includes the total prorated facility cost and the total expected operational cost. At first, a number of facilities have been built and their locations are denoted by set J^*^. Building a facility at location *j* ∈ J^*^ (or facility *j* as short) needs a fixed investment equivalent to an prorated cost of *f*_*j*_. Therefore, the total facility construction cost is formulated by

$$C^{\text{F}}=\sum_{j\in J^{*}}f_{j}$$ (1)

**Fig 1:**
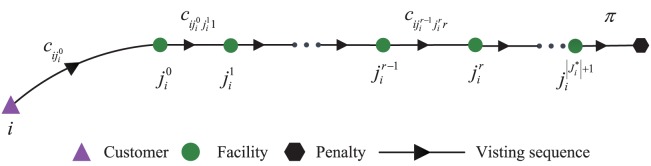
Visiting sequence for arbitrary customer *i*.

Then we try to formulate the total expected operational cost that is constituted by the transportation cost and penalty cost. For each customer i∈I, her demand is denoted by *λ*_*i*_. The subset $J_{i}^{*}\subseteq J^{*}$ denotes the set of the facilities which are assigned to service customer *i*. We rank the facilities in $J_{i}^{*}$ as $\left\{j_{i}^{0},j_{i}^{1},\cdots ,j_{i}^{r},\cdots ,j_{i}^{\left|J_{i}^{*}\right|+1}\right\}$. We define $j_{i}^{r}$ as the level-*r* assigned facility for customer *i*. Facility $j_{i}^{0}$ is treated as customer *i*'s primary facility and the other facilities in $J_{i}^{*}$ are treated as her backup facilities. *d*_*ij*_ denotes the Euclidean distance from customer *i* to facility *j*. *d*_*jj*'_ denotes the Euclidean distance from facility *j* to another facility *j*'. We define $c_{ij_{i}^{0}}$ as the unit-demand transportation cost (which could be the product of $d_{ij_{i}^{0}}$ and a transportation rate *α*). We define $c_{ij_{i}^{r-1}j_{i}^{r}r}$ as the unit-demand transportation cost when customer *i* goes to facility $j_{i}^{r}$ from $j_{i}^{r-1}$ (which could similarly be *α* times distance $d_{j_{i}^{r-1}j_{i}^{r}}$). For each facility *j* ∈ J^*^, its disruption probability is denoted by *q*_*j*_, which apparently depends on location *j*. If customer *i* gives up looking for the service, she will bear a unit-demand penalty cost which is denoted by *π*. Fig 1 illustrates the visiting sequence for arbitrary customer *i*.

For each customer i∈I, she goes to her primary facility $j_{i}^{0}$ at first. If facility $j_{i}^{0}$ is operating, she obtains the service and her transportation cost is formulated by

$$\lambda_{i}\left(1-q_{j_{i}^{0}}\right)c_{ij_{i}^{0}}.$$ (2)

Otherwise, she goes to her next level facility $j_{i}^{1}$ from her present location. If the facility $j_{i}^{1}$ is operating, she obtains the service and her transportation cost is formulated by

$$\lambda_{i}q_{j_{i}^{0}}\left(1-q_{j_{i}^{1}}\right)\left(c_{ij_{i}^{0}}+c_{ij_{i}^{0}j_{i}^{1}1}\right).$$ (3)

Otherwise, she goes to her next level facility $j_{i}^{2}$. The process will be going on until she reaches facility $j_{i}^{\left|J_{i}^{*}\right|+1}$. If this facility is operating, she obtains the service and her transportation cost is formulated by

$$\lambda_{i}\left(\prod_{r=0}^{\left|J_{i}^{*}\right|}q_{j_{i}^{r}}\right)\left(1-q_{j_{i}^{R}}\right)\left(c_{ij_{i}^{0}}+\sum_{r=1}^{\left|J_{i}^{*}\right|+1}c_{ij_{i}^{r-1}j_{i}^{r}r}\right).$$ (4)

Otherwise, she gives up the service and bears the penalty cost which is formulated as

$$\lambda_{i}\left(\prod_{r=0}^{\left|J_{i}^{*}\right|+1}q_{j_{i}^{r}}\right)\pi .$$ (5)

And she also has the transportation cost formulated as

$$\lambda_{i}\left(\prod_{r=0}^{\left|J_{i}^{*}\right|+1}q_{j_{i}^{r}}\right)\left(c_{ij_{i}^{0}}+\sum_{r=1}^{\left|J_{i}^{*}\right|+1}c_{ij_{i}^{r-1}j_{i}^{r}r}\right).$$ (6)

Therefore, the expected transportation cost for customer *i* is formulated by

$$\lambda_{i}\left(\left(1-q_{j_{i}^{0}}\right)c_{ij_{i}^{0}}+\sum_{r=1}^{\left|J_{i}^{*}\right|+1}\left(\left(\prod_{r'=0}^{r-1}q_{j_{i}^{r'}}\right)\left(1-q_{j_{i}^{r}}\right)\left(c_{ij_{i}^{0}}+\sum_{r'=1}^{r}c_{ij_{i}^{r'-1}j_{i}^{r'}r'}\right)\right)+\left(\prod_{r=0}^{\left|J_{i}^{*}\right|+1}q_{j_{i}^{r}}\right)\left(c_{ij_{i}^{0}}+\sum_{r=1}^{\left|J_{i}^{*}\right|+1}c_{ij_{i}^{r-1}j_{i}^{r}r}\right)\right).$$ (7)

This formulation can be simplified and rewritten as

$$\lambda_{i}\left(c_{ij_{i}^{0}}+\sum_{r=1}^{\left|J_{i}^{*}\right|+1}\left(\prod_{r'=0}^{r-1}q_{j_{i}^{r'}}\right)c_{ij_{i}^{r-1}j_{i}^{r}r}\right).$$ (8)

According to Eqs (5) and (8), we can obtains the total expected operational cost formulated as

$$C^{\text{O}}=\sum_{i\in I}\lambda_{i}\left(c_{ij_{i}^{0}}+\sum_{r=1}^{\left|J_{i}^{*}\right|+1}\left(\prod_{r'=0}^{r-1}q_{j_{i}^{r'}}\right)c_{ij_{i}^{r-1}j_{i}^{r}r}+\left(\prod_{r=0}^{\left|J_{i}^{*}\right|+1}q_{j_{i}^{r}}\right)\pi \right),$$ (9)

And thus the total expected system cost is formulated by

$$C=C^{\text{F}}+C^{\text{O}}=\sum_{j\in J^{*}}f_{j}+\sum_{i\in I}\lambda_{i}\left(c_{ij_{i}^{0}}+\sum_{r=1}^{\left|J_{i}^{*}\right|+1}\left(\prod_{r'=0}^{r-1}q_{j_{i}^{r'}}\right)c_{ij_{i}^{r-1}j_{i}^{r}r}+\left(\prod_{r=0}^{\left|J_{i}^{*}\right|+1}q_{j_{i}^{r}}\right)\pi \right).$$ (10)

**Fig 2:**
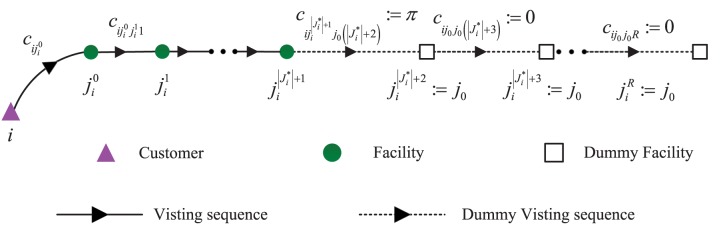
Visiting sequence of assigned facilities with padding dummy facility.

In order to formulate the total expected system cost conveniently, we reformulate the operational costs associated with different customers into a unified form (which facilitates formulation of the location design model in the next section). Fig 2 shows the unified form of visiting sequence for arbitrary customer *i*. In this figure, the triangle denotes the customer, the circle denotes the actual built facilities, and the square denotes the dummy facility defined as follows. We introduce a dummy facility location *j*_0_ such that customer *i*'s loss of service is equivalently represented by visiting the dummy facility *j*_0_ from the last actual facility $j_{i}^{\left|J_{i}^{*}\right|+1}$. Then we expand the set $J_{i}^{*}$ to ${\bar{J}}_{i}^{*}$ by padding it with *j*_0_ to length *R*, where *R* is a sufficiently large number. We usually set $R=\max{\left\{\left|J_{i}^{*}\right|\right\}}_{i\in I}+2$. The facilities in set ${\bar{J}}_{i}^{*}$ can be ranked as $\left\{j_{i}^{0},j_{i}^{1},\cdots ,j_{i}^{r},\cdots ,j_{i}^{R}\right\}$. When $r>\left|J_{i}^{*}\right|+1$, we set $j_{i}^{r}=j_{0}$. We also define $c_{ij_{0}}=\pi ,\forall i\in I$, $c_{ijj_{0}r}=\pi ,\forall i\in I,j\in J_{i}^{*},r=1,2,\cdots ,R$ and $c_{ij_{0}j_{0}r}=0,\forall i\in I,r=1,2,\cdots ,R$. Then the Eq (9) can be rewritten as

$$C^{\text{O}}=\sum_{i\in I}\lambda_{i}\left(c_{ij_{i}^{0}}+\sum_{r=1}^{R}\left(\prod_{r'=0}^{r-1}q_{j_{i}^{r'}}\right)c_{ij_{i}^{r-1}j_{i}^{r}r}\right).$$ (11)

Accordingly, the total expected system cost is formulated as below

$$C=\sum_{j\in J^{*}}f_{j}+\sum_{i\in I}\lambda_{i}\left(c_{ij_{i}^{0}}+\sum_{r=1}^{R}\left(\prod_{r'=0}^{r-1}q_{j_{i}^{r'}}\right)c_{ij_{i}^{r-1}j_{i}^{r}r}\right)$$ (12)

In Eq (12), the pattern of disruption probabilities ${\left\{q_{j_{i}^{r}}\right\}}_{\forall j_{i}^{r}}$ captures site-dependence of facility disruptions, and thus mark the major contribution of the proposed model that is formulated in detail in the next section.

### Reliable facility location design model

The investigated reliable facility location design problem aims is to find the optimal facility locations among all candidates to minimize the total cost including the one-time facility built cost and the long-term operational cost. We define the set J to denote all candidate facility locations. According to the description of the system framework in the previous section, we formulate this problem as follows

$$\underset{J^{*}\subseteq J,{\left\{J_{i}^{*}\right\}}_{i\in I}}{\min}C=\sum_{j\in J^{*}}f_{j}+\sum_{i\in I}\lambda_{i}\left(c_{ij_{i}^{0}}+\sum_{r=1}^{R}\left(\prod_{r'=0}^{r-1}q_{j_{i}^{r'}}\right)c_{ij_{i}^{r-1}j_{i}^{r}r}\right).$$ (13)

Model (13) is however highly nonlinear and might not be easy to solve efficiently. The remainder of this section will propose an equivalent linear integer programming (LIP) model that is suitable for commercial solvers and systematic algorithms. We define decision variables to specify the location decision $Y={\left\{y_{j}\right\}}_{j\in J}$, where

$$y_{j}=\{\begin{array}{cc} 1, & \text{if}\;\text{facility}\;j\;\text{is}\;\text{open}\;(\text{or}\;j\in J^{*});\\ 0, & \text{otherwise}\;(\text{or}\;j\notin J^{*}). \end{array}$$ (14)

Then the annual total facility construction cost (1) can be equivalently formulated as

$$\sum_{j\in J}f_{j}y_{j}$$ (15)

For notation convenience, we define

$$\bar{J}≔J\cup \left\{j_{0}\right\},J_{j}^{+}≔\{\begin{array}{cc} \bar{J} & j=j_{0};\\ J\backslash \left\{j\right\}, & j\neq j_{0}, \end{array}J_{j}^{-}≔\{\begin{array}{cc} j_{0} & j=j_{0};\\ \bar{J}\backslash \left\{j\right\} & j\neq j_{0}, \end{array},\forall j\in \bar{J},$$ (16)

where $J_{j}^{+}$ and $J_{j}^{-}$ are the candidate facility locations that can be visited before and after facility *j*, respectively.

We define two sets of auxiliary decision variables to specify facility assignments, $X={\left\{x_{ij}\right\}}_{i\in I,j\in \bar{J}}$ and $X'={\left\{x_{ijj'r}\right\}}_{i\in I,j\in \bar{J},j'\in J_{j}^{-},r=1,2,\cdots ,R}$, where

$$x_{ij}=\{\begin{array}{cc} 1, & \text{if}\;\text{customer}\;i\;\text{is}\;\text{assigned}\;\text{to}\;\text{faiclity}\;j\;\text{at}\;\text{rank}\;0;\\ 0, & \text{otherwise}, \end{array}$$ (17)

and

$$x_{ijj'r}=\{\begin{array}{cc} 1, & \text{if}\;i\;\text{is}\;\text{assigned}\;\text{to}\;j\;\text{at}\;\text{rank}\;r\text{-}1\;\text{and}\;\text{to}\;j'\;\text{at}\;\text{rank}\;r,\;\forall r=1,2,\cdots ,R;\\ 0, & \text{otherwise}. \end{array}$$ (18)

Because $\left(\prod_{r'=0}^{r-1}q_{j_{i}^{r'}}\right)$ is variable with the different customer’s visiting sequence, we define the set of probability variables $P={\left\{p_{ijj'r}\right\}}_{i\in I,j\in \bar{J},j'\in J_{j}^{-},r=1,2,\cdots ,R}$. In this set, *p*_*ijj*'*r*_ is the probability that customer *i* visits facility *j*' at level *r* after visiting facility *j*.

With these definitions, the total expected operational cost (11) can be rewritten as

$$\sum_{i\in I}\lambda_{i}\sum_{j\in \bar{J}}\left(c_{ij}x_{ij}+\sum_{j'\in J_{j}^{-}}\sum_{r=1}^{R}c_{ijj'r}p_{ijj'r}x_{ijj'r}\right).$$ (19)

With formulation (15) and (19), the reliable facility location design problem can be formulated into an LIP model as follows:

$$\underset{X,X',Y,P}{\min}\sum_{j\in J}f_{j}y_{j}+\sum_{i\in I}\lambda_{i}\sum_{j\in \bar{J}}\left(c_{ij}x_{ij}+\sum_{j'\in J_{j}^{-}}\sum_{r=1}^{R}c_{ijj'r}p_{ijj'r}x_{ijj'r}\right)$$ (20)

subject to

$$x_{ij}+\sum_{j'\in J_{j}^{+}}\sum_{r=1}^{R}x_{ij'jr}\leq y_{j},\forall i\in I,j\in J$$ (21)

$$\sum_{j\in J}x_{ij}=1,\forall i\in I$$ (22)

$$x_{ij}=\sum_{j'\in J_{j}^{-}}x_{ijj'1},\forall i\in I,j\in \bar{J}$$ (23)

$$\sum_{j'\in J_{j}^{+}}x_{ij'j\left(r-1\right)}=\sum_{j'\in J_{j}^{-}}x_{ijj'r},\forall i\in I,j\in \bar{J},r=2,3,\cdots ,R$$ (24)

$$\sum_{j\in \bar{J}}x_{ijj_{0}R}=1,\forall i\in I$$ (25)

$$p_{ijj'1}=q_{j},\forall i\in I,j\in \bar{J},j'\in J_{j}^{-}$$ (26)

$$p_{ijj'r}=q_{j}\sum_{j'\in J_{j}^{+}}p_{ij'j\left(r-1\right)}x_{ij'j\left(r-1\right)},\forall i\in I,j\in \bar{J},j'\in J_{j}^{-},r=2,3,\cdots ,R$$ (27)

$$y_{j}\in \{0,1\},\;\forall j\in J$$ (28)

$$x_{ij}\in \left\{0,1\right\},\forall i\in I,j\in \bar{J}$$ (29)

$$x_{ijj'r}\in \left\{0,1\right\},\forall i\in I,j\in ,j'\in J_{j}^{-},r=1,2,\cdots ,R$$ (30)

Objective function Eq (20) aims to find the minimum system cost and the optimal facility location scheme accordingly. Constraints (21) prohibit an assignment to an unbuilt facility or multiple assignments of the same built facility to a customer. Constraints (22) ensure that each customer must be assigned to only one primary facility. Constraints (23) and (24) mean that a customer’s move at the level *r* facility always starts from her level (*r*−1) assigned facility. Constraints (25) ensure that the customer will finally move to the dummy facility so as to correctly account for the penalty cost. Constraints (26) and (27) illustrate the transformation between the facilities in the adjacent level. Constraints (28)-(30) postulate integral constraints to all decision variables.

Model (20)-(30) is a concise mathematic programming model for the reliable facility location design with site-independent disruptions under imperfection information. However, the model is still nonlinear even without the integer constraints. Note that the only nonlinear terms in this model are $p_{ijj'r}x_{ijj'r},\forall i\in I,j\in \bar{J},j'\in J_{j}^{-},r=1,2,\cdots ,R$, each of which is the product of a continuous variable and a binary variable. We apply the linearization technique used in the previous literature [7, 31], to replace each *p*_*ijj*'*r*_*x*_*ijj*'*r*_ term with a new variable *w*_*ijj*'*r*_. Then a set of new constraints are added to Model (20)-(30) to enforce $w_{ijj'r}≔p_{ijj'r}x_{ijj'r},\forall i\in I,j\in \bar{J},j'\in J_{j}^{-},r=1,2,\cdots ,R$, as follows:

$$w_{ijj'r}\leq p_{ijj'r},\forall i\in I,j\in \bar{J},j'\in J_{j}^{-},r=1,2,\cdots ,R$$ (31)

$$w_{ijj'r}\leq x_{ijj'r},\forall i\in I,j\in \bar{J},j'\in J_{j}^{-},r=1,2,\cdots ,R$$ (32)

$$w_{ijj'r}\geq 0,\forall i\in I,j\in \bar{J},j'\in J_{j}^{-},r=1,2,\cdots ,R$$ (33)

$$w_{ijj'r}\geq p_{ijj'r}+x_{ijj'r}-1,\forall i\in I,j\in \bar{J},j'\in J_{j}^{-},r=1,2,\cdots ,R$$ (34)

The linearize formulation of model (20)-(30) is stated below:

$$\underset{X,X',Y,P}{\min}\sum_{j\in J}f_{j}y_{j}+\sum_{i\in I}\lambda_{i}\sum_{j\in \bar{J}}\left(c_{ij}x_{ij}+\sum_{j'\in J_{j}^{-}}\sum_{r=1}^{R}c_{ijj'r}w_{ijj'r}\right)$$ (35)

subject to

$$x_{ij}+\sum_{j'\in J_{j}^{+}}\sum_{r=1}^{R}x_{ij'jr}\leq y_{j},\forall i\in I,j\in J$$ (36)

$$\sum_{j\in J}x_{ij}=1,\forall i\in I$$ (37)

$$x_{ij}=\sum_{j'\in J_{j}^{-}}x_{ijj'1},\forall i\in I,j\in \bar{J}$$ (38)

$$\sum_{j'\in J_{j}^{+}}x_{ij'j\left(r-1\right)}=\sum_{j'\in J_{j}^{-}}x_{ijj'r},\forall i\in I,j\in \bar{J},r=2,3,\cdots ,R$$ (39)

$$\sum_{j\in \bar{J}}x_{ijj_{0}R}=1,\forall i\in I$$ (40)

$$w_{ijj'r}\leq p_{ijj'r},\forall i\in I,j\in \bar{J},j'\in J_{j}^{-},r=1,2,\cdots ,R$$ (41)

$$w_{ijj'r}\leq x_{ijj'r},\forall i\in I,j\in \bar{J},j'\in J_{j}^{-},r=1,2,\cdots ,R$$ (42)

$$w_{ijj'r}\geq 0,\forall i\in I,j\in \bar{J},j'\in J_{j}^{-},r=1,2,\cdots ,R$$ (43)

$$w_{ijj'r}\geq p_{ijj'r}+x_{ijj'r}-1,\forall i\in I,j\in \bar{J},j'\in J_{j}^{-},r=1,2,\cdots ,R$$ (44)

$$p_{ijj'1}=q_{j},\forall i\in I,j\in \bar{J},j'\in J_{j}^{-}$$ (45)

$$p_{ijj'r}=q_{j}\sum_{j'\in J_{j}^{+}}w_{ij'j\left(r-1\right)},\forall i\in I,j\in \bar{J},j'\in J_{j}^{-},r=2,3,\cdots ,R$$ (46)

$$y_{j}\in \{0,1\},\;\forall j\in J$$ (47)

$$x_{ij}\in \left\{0,1\right\},\forall i\in I,j\in \bar{J}$$ (48)

$$x_{ijj'r}\in \left\{0,1\right\},\forall i\in I,j\in \bar{J},j'\in J_{j}^{-},r=1,2,\cdots ,R$$ (49)

Although this problem is an NP-hard problem, from our computational experiments, the linear structure of Model (35)-(49) allows it to be solved by existing state-of-the-art linear programming integer commercial solvers (e.g., Gurobi, CPLEX) to an exact or near-optimum solution in a reasonable time. The following case study illustrates this with problem instances of reasonable sizes.

## Case study

In this section, we will test the proposed model against a number of problem instances and draw interesting managerial insights into optimal facility layouts and parameter sensitivity. These problem instances are generated with one set of real-world data. The data set is derived by Daskin [2] from 1990 population and housing census data: the 49-node set including 48 US state capitals and Washington, DC. We process the data in a way similar to Snyder and Daskin [6] to obtain the model parameters. These nodes in the 49-node set are the locations for both candidate facilities and customers. Demands ${\left\{\lambda_{i}\right\}}_{i\in I}$ are set to the corresponding state population divided by 10^5^ for the 49-node set. The fixed annual cost *f*_*j*_ at each city is set to the median home value in the city. To factor in detours due to roadway networks, the distance between two locations is calculated by multiplying a coefficient of 1.2 to their great circle distance [32]. We set the facility disruption probability $q_{j}=\rho e^{-f_{j}/200000},\forall j\in J$ where coefficient *ρ* is used to control the overall magnitude of the disruption probabilities. This disruption probability setting means a higher facility construction cost results a lower facility disruption probability. We set the default values of the key parameters as: *α* = 1, *ρ* = 0.1, *π* = 10000 and *R* = 4. Note that these default values may vary in certain instances. All problem instances are solved by a commercial programming solver, Gurobi, on a PC with 3.4 GHz CPU and 16 GB RAM.

### Optimal facility layouts

In this subsection, we discuss the model performance and the optimal facility layouts in different problem instances with the default parameter values. According to the populations of the 49-node set, we construct three data sets that include the first 15, 25 and 35 populous US state capitals respectively.

Table 1 shows the model performance in the different instances with a solution time limit of 3600s. In this table, we can see that most of instances except the last three instances can be solved by the solver Gurobi with a small optimal gap (no more than 5%), which shall suffice most engineering needs. This verifies the practical applicability of the proposed model in solving network infrastructure planning with site-dependent disruptions under imperfect information. However, the solution time and the optimality gap increase with the disruption probability for instances with same scale and increase with the instance scale for instances with the same disruption probability. The solution times of several instances reach the time limitation (3600 seconds). And the optimality gap is even up to 20% in the 49-node set with *ρ* = 0.3. It indicates that the off-the-shelf solver, Gurobi, has limitations when dealing with the large-scale or high disruption probability problem instances. Therefore, a customize algorithm should be proposed if we need solve large-scale problems efficiently, which is however out of the scope of this study and will be investigated in future study. In another aspect, we find that a few instances have the same optimal facility layouts, i.e., 25-node set and 35-node set with *ρ* = 0.05, 35-node set and 49-node set with *ρ* = 0.1, 15-node set with *ρ* = 0.05 and *ρ* = 0.1, 25-node with *ρ* = 0.05 and *ρ* = 0.1. These findings indicate that the optimal facility layouts have a good robust performance in resisting facility disruptions and across small variations of the instance size.

**Table 1:** Model performance in the different instances.

Node	*ρ*	Best objective	Best bound	Gap(%)	Locations	Time(s)
15	0.05	643425.58	6.43383.59.	0.0065	1,3,4,5,6,8	8
25	0.05	823126.09	823124.37	0.0002	1,3,5,6,8,22	60
35	0.05	952731.61	950545.85	0.2294	1,3,5,6,8,22	235
49	0.05	1019874.54	1.016689.51	0.3123	1,3,5,7,22,30	609
15	0.1	692638.02	692611.80	0.0038	1,3,4,5,6,8	13
25	0.1	882565.35	882483.94	0.0092	1,3,5,6,8,22	170
35	0.1	1008318.81	1003288.80	0.4989	1,3,5,6,7,22,29	534
49	0.1	1076761.78	1069289.67	0.6939	1,3,5,6,7,22,29	2931
15	0.2	804767.21	796746.33	0.9967	1,3,4,5,6,7	834
25	0.2	1014739.72	998609.18	1.5896	1,3,5,6,7,22	3600
35	0.2	1130801.61	1096305.83	3.0506	1,3,5,6,9,14,22,29	3600
49	0.2	1201601.49	1152557.85	4.0815	1,2,3,5,6,14,22,29	3600
15	0.3	941342.42	896616.27	4.7513	1,3,4,5,6,7,9	3600
25	0.3	1161838.52	1076286.93	7.3635	1,3,5,6,9,14,22,24	3600
35	0.3	1286516.19	1149413.08	10.6569	1,3,5,6,9,14,22,29,31	3600
49	0.3	1515634.15	1210586.69	20.1267	1,3,5,6,9,14,22,29,31	3600

**Fig 3:**
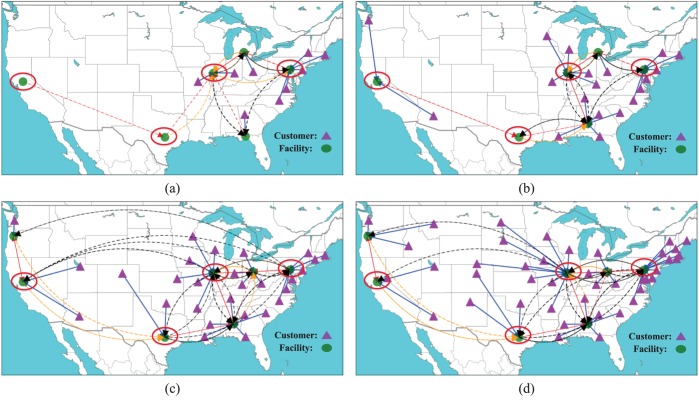
Optimal facility layouts in different scale instances. (a) 15-node set. (b) 25-node set. (c) 35-node set. (d) 49-node set.

Fig 3 shows the optimal facility layouts and customer-facility assignments marked by different color lines for instances at different scales with *ρ* = 0.1. In these figures, the circles denote built facilities, and the triangles denote customers. The blue solid arrows mark level-1 customer-facility assignments, the red dashed arrows mark level-2 customer-facility assignments, the yellow dashed arrows mark level-3 customer-facility assignments and the black dashed arrows mark level-4 customer-facility assignments. We see that with the increase of instance scale, more facilities are constructed to minimize the total system cost. However, several facilities in the red circle do not change in different scale instances. It indicates that these facilities play a key role in the facility location layouts. We can reinforce these facilities, reducing their disruption probabilities to enhance the system reliability. In Table 1, we know that the 35-node set and 49-node set with *ρ* = 0.1 have the same facility layouts. However, from Fig 3(C) and 3(D), we find that these two instances at different levels have the different customer-facility assignments. It indicates that we should adjust the customer-facility assignments for different conditions although the facility location layout remains unchanged.

**Fig 4:**
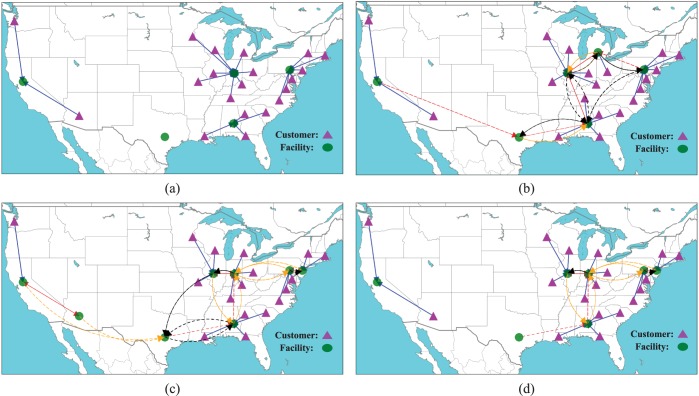
Optimal facility layouts under different disruption probability rate and penalty rate. (a) *ρ* = 0, *π* = 10000. (b) *ρ* = 0.1, *π* = 10000. (c) *ρ* = 0.3, *π* = 10000. (d) *ρ* = 0.3, *π* = 1000.

Fig 4 shows the optimal facility layouts and customer-facility assignments under different *ρ* and *π* values by using the 25-node data set. In Fig 4(A), when facilities do not suffer disruptions, the problem reduces to a classic uncapacitated fixed location problem where a customer is always served by her nearest operational facility. We consider this facility location solution as the benchmark solution. In Fig 4(B), as probability rate *ρ* increases to 0.1, one additional facility is built so that a customer has more convenient access to both primary and backup facilities. In Fig 4(C), as probability rate *ρ* increases to 0.3, two more facilities are built to enhance the accessibility of backup services. Fig 4(D) shows the optimal facility layouts when the penalty cost is decreased into a smaller value, i.e.,10^3^. We see that one facility is removed and a number of high level customer-facility assignments disappear. This means that customers only travel to their nearby facilities within a certain acceptable distance or completely give up the service if the service is not worth the transportation cost. In other word, a larger penalty cost tends to force customers to try more backup facilities at higher level assignments so as to further reduce the risk of losing the service.

**Fig 5:**
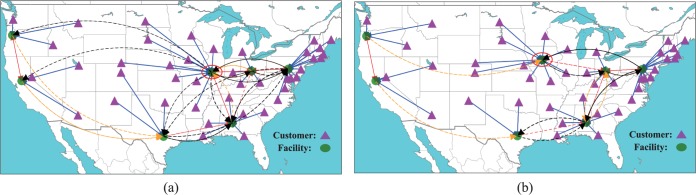
Optimal facility layouts under site-dependent and identical independent disruption. (a) site-dependent (*ρ* = 0.1). (b) independent (*p* = 0.07).

Fig 5 compares the optimal facility layout under site-dependent disruptions with that under identical disruption probabilities by using the 49-node data set. In order to make the disruption probabilities as close as possible in the two cases, the disruption probability in the identical disruption scenario is set equal to the average value of the disruption probabilities in the site-dependent case, i.e., $p=\sum_{j\in J}q_{j}/\left|J\right|$. In this figure, we can see that compared to the identical independent disruption case, one facility is built at a different location to better support the other facilities under site-dependent disruptions (as highlighted by the red circle in Fig 5). Interestingly, the customer-facility assignments under site-dependent disruption are more complex than those under identical disruptions, and the changes are mainly that customers are more likely to be assigned to facilities with lower disruption probabilities.

### Sensitivity analysis

In this subsection, we discusses the sensitivity of the optimal results to parameters *α*, *π*, *ρ*, and *R* for the 25-node data set. In order to illustrate the benefit of backup facility, Table 2 shows the optimal facility locations and the corresponding cost components for instances with *R* ranging from 1 through 10. The instance with *R* = 1 indicates that the customers can just obtain the service from the primary facility without the backup facility. In this table, we can see that the total system cost decreases 54% if we pad one backup facility for all customers. In other word, padding one backup facility brings a net social benefit about 54% of the maximum potential. With the increase of *R*, more backup facilities are padded. Both the construction cost and the penalty cost decrease. The transportation cost increases because the customer needs visit more facilities to obtain the service. As a result, the total system cost continues going down. Asymptotically, padding sufficient backup facilities can reduce the total system cost to 41% of that when only the primary facility is allowed (or when *R* = 1). This indicates that padding backup facilities can bring a net social benefit about 59% of the maximum potential compared with only the primary facility. This suggests that a substantial saving can be achieved by allowing for providing backup facilities when the system is properly designed, even under imperfect information. We also find that the total system cost change is less than 0.01% when *R* is greater than or equal to 4. Therefore, we choose the value of *R* equal to 4 for the most instances.

**Table 2:** Sensitivity to *R*.

*R*	Locations	Construction cost	Transportation cost	Penalty cost	Total system cost
1	1,3,4,6,19	4.59E+05	4.63E+05	1.24E+06	2.16E+06
2	1,3,5,6,7,22	4.14E+05	4.63E+05	1.08E+05	9.85E+05
3	1,3,5,6,8,22	3.97E+05	4.85E+05	8.78E+03	8.90E+05
4	1,3,5,6,8,22	3.97E+05	4.85E+05	6.59E+02	8.83E+05
5	1,3,5,6,8,22	3.97E+05	4.85E+05	4.63E+01	8.82E+05
6	1,3,5,6,8,22	3.97E+05	4.85E+05	0.00E+00	8.82E+05
7	1,3,5,6,8,22	3.97E+05	4.85E+05	0.00E+00	8.82E+05
8	1,3,5,6,8,22	3.97E+05	4.85E+05	0.00E+00	8.82E+05
9	1,3,5,6,8,22	3.97E+05	4.85E+05	0.00E+00	8.82E+05
10	1,3,5,6,8,22	3.97E+05	4.85E+05	0.00E+00	8.82E+05

**Fig 6:**
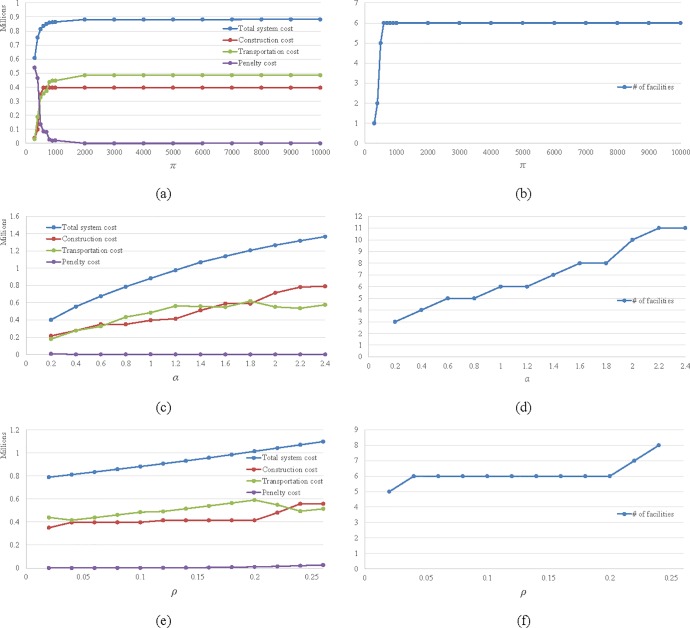
Sensitivity analysis.

Fig 6 shows the sensitivity analysis results on how the optimal solution changes with parameter values. The default parameter values are *α* = 1, *ρ* = 0.1, *π* = 10000, and we vary one parameter at a time.

Fig 6(A) and 6(B) show the effect of penalty rate *π* on the cost components and the number of facilities respectively. When *π* is small, only a few of facilities are built because a customer just obtains the service from the nearby facility and farther facilities is not worth building. As *π* is increasing, customers are more intended to look for the service from backup facilities instead of easily giving up for the service. Therefore, additional facilities should be built to eliminate the unnecessary penalty cost. We see this regular in Fig 6(A) where the construction cost and transportation cost increase to high level sharply as the increase of *π*. Although *π* is increasing, the penalty cost still decreases to a low level quickly because the probability of giving up the service decreases exponentially. When *π* is more than 2000, the system seems to already have sufficient facilities and stay at a steady and reliable state. In this state, the transportation cost and construction cost remain the same and the total penalty cost increases at a low rate. These results provide a very useful guidance for designing a reliable system to be robust against the change of penalty rate.

Fig 6(C) and 6(D) show how the cost components and the number of facilities, respectively, change as transportation rate *α* increases. In Fig 6, as we can see, the construction cost, the transportation cost and the facility number increase with the increase of *α* while the penalty cost remains at a low value across all *α* values. It is intuitive that the increase of transportation rate results in the rise of the total transportation cost. In order to reduce the increase rate of the transportation cost, more facilities should be built consequentially. Since there are enough built facilities to be assigned to customers at all levels to satisfy the customers' demand, the penalty cost always occurs in the last level and remains at a low value regardless of the magnitude of *α*.

Fig 6(E) and 6(F) show the effects from increasing disruption probability rate *ρ*. We see that as the facility disruption risks increase, the probability that customers suffer the penalty increases and thus it causes the penalty cost to increase. In the meanwhile, the transportation cost also increases because customers have a higher possibility to access their backup facilities. Once the increment of the transportation cost is worth the adjustment of facility layout, alternative facilities or additional facilities will be built (see Fig 6(F)) and the construction cost will increase (see Fig 6(E)). Then the customers are likely to access the service with relatively low transportation expenditures and the transportation cost will decrease for a while (see Fig 6(E)). On the other hand, for a certain facility layout, the system has a certain robust performance when the disruption probability changes (see Fig 6(E), where the construction cost does not change much during a large range of *ρ*). However, the total system cost is still increasing gradually as the disruption probability increases.

## Conclusion

This paper proposed a reliable facility location design model that allows each facility to be disrupted at a site-dependent probability. The model addresses the imperfect information context: a customer uses the “trial-or-error” strategy to search the service due to the lack of real time information of facility states. The formulated optimization model determines the optimal facility location and facility-customer assignments that minimize the total system cost including the facility construction investment and the expected transportation and penalty costs. A linearization technique is applied to transform the nonlinear programming model into a linear programming model so that this model is able to be solved by available commercial solvers. We tested the performance of the proposed model with a number of numerical instances. The results indicated that most of the instances can be efficiently solved to obtain the optimum solution with a tight optimality gap. We also made a series of sensitivity analysis to illustrate how the optimal solution changes with different parameter values. We found that padding backup facilities for the customers can significantly reduce the total system cost, and there exists a steady state of the reliable design where the system becomes robust against further increase of loss-of-service penalty. With the increase of transportation cost rate, redesigning the facility location layouts can reduce the total system cost compared with the previous facility layouts.

This study focuses on facility location problems with the site-dependent disruption under imperfect information. It will be interesting to study the correlated or interdependent disruptions for facility location problems under imperfect information. Our proposed model is solved by a commercial solver for median or small scale instances with a reasonable gap. In order to deal with large-scale facility location problem instances, a customized algorithm will be needed to cut down the computational time and enhance the solution quality. In addition, we assume that the customer demand is constant in our study. In order to capture stochastic systems with relatively volatile demand, it will be interesting to investigate how to integrate the dynamics and uncertain demand to this model framework.

## Supporting information

S1 Dataset Data for case study. (DOCX)
