# Supplementary material for: A reliable facility location design model with site-dependent disruption in the imperfect information context
Source: PLoS One. 2017 May 9;12(5):e0177104. doi: 10.1371/journal.pone.0177104 (PMC5423640; doi:10.1371/journal.pone.0177104)
Supplement: S1 Dataset — (DOCX) [file pone.0177104.s001.docx]

**Data for case study**

| City | ST | Pop. Rank | Longitude | Latitude | State Pop. | Fixed Cost |
| --- | --- | --- | --- | --- | --- | --- |
| Sacramento | CA | 1 | -121.467 | 38.567 | 29760021 | 67900 |
| Albany | NY | 2 | -73.799 | 42.666 | 17990455 | 38400 |
| Austin | TX | 3 | -97.751 | 30.306 | 16986510 | 62200 |
| Tallahassee | FL | 4 | -84.281 | 30.457 | 12937926 | 77100 |
| Harrisburg | PA | 5 | -76.885 | 40.276 | 11881643 | 112400 |
| Springfield | IL | 6 | -89.645 | 39.781 | 11430602 | 96600 |
| Columbus | OH | 7 | -82.987 | 39.989 | 10847115 | 67900 |
| Lansing | MI | 8 | -84.554 | 42.709 | 9295297 | 113000 |
| Trenton | NJ | 9 | -74.764 | 40.223 | 7730188 | 61700 |
| Raleigh | NC | 10 | -78.659 | 35.822 | 6628637 | 67700 |
| Atlanta | GA | 11 | -84.423 | 33.763 | 6478216 | 123900 |
| Richmond | VA | 12 | -77.475 | 37.531 | 6187358 | 60800 |
| Boston | MA | 13 | -71.018 | 42.336 | 6016425 | 60300 |
| Indianapolis | IN | 14 | -86.146 | 39.776 | 5544159 | 63200 |
| Jefferson City | MO | 15 | -92.19 | 38.572 | 5117073 | 161400 |
| Madison | WI | 16 | -89.388 | 43.08 | 4891769 | 72600 |
| Nashville-Davidson | TN | 17 | -86.785 | 36.172 | 4877185 | 59200 |
| Olympia | WA | 18 | -122.894 | 47.042 | 4866692 | 68700 |
| Annapolis | MD | 19 | -76.503 | 38.972 | 4781468 | 79500 |
| St. Paul | MN | 20 | -93.104 | 44.948 | 4375099 | 77800 |
| Baton Rouge | LA | 21 | -91.126 | 30.449 | 4219973 | 64200 |
| Montgomery | AL | 22 | -86.284 | 32.354 | 4040587 | 79000 |
| Frankfort | KY | 23 | -84.865 | 38.191 | 3685296 | 48800 |
| Phoenix | AZ | 24 | -112.071 | 33.543 | 3665228 | 133800 |
| Columbia | SC | 25 | -80.886 | 34.039 | 3486703 | 88700 |
| Denver | CO | 26 | -104.873 | 39.768 | 3294394 | 54600 |
| Hartford | CT | 27 | -72.684 | 41.766 | 3287116 | 66000 |
| Oklahoma City | OK | 28 | -97.513 | 35.467 | 3145585 | 71200 |
| Salem | OR | 29 | -123.022 | 44.925 | 2842321 | 94100 |
| Des Moines | IA | 30 | -93.617 | 41.577 | 2776755 | 101800 |
| Jackson | MS | 31 | -90.208 | 32.321 | 2573216 | 115800 |
| Topeka | KS | 32 | -95.692 | 39.038 | 2477574 | 48400 |
| Little Rock | AR | 33 | -92.354 | 34.722 | 2350725 | 72400 |
| Charleston | WV | 34 | -81.63 | 38.351 | 1793477 | 61500 |
| Salt Lake City | UT | 35 | -111.93 | 40.777 | 1722850 | 71300 |
| Lincoln | NE | 36 | -96.688 | 40.816 | 1578385 | 99000 |
| Santa Fe | NM | 37 | -105.954 | 35.679 | 1515069 | 49500 |
| Augusta | ME | 38 | -69.73 | 44.331 | 1227928 | 67200 |
| Carson City | NV | 39 | -119.743 | 39.148 | 1201833 | 70900 |
| Concord | NH | 40 | -71.56 | 43.232 | 1109252 | 59500 |
| Boise City | ID | 41 | -116.226 | 43.607 | 1006749 | 74400 |
| Providence | RI | 42 | -71.42 | 41.822 | 1003464 | 54900 |
| Helena | MT | 43 | -112.02 | 46.597 | 799065 | 75200 |
| Pierre | SD | 44 | -100.322 | 44.373 | 696004 | 66600 |
| Dover | DE | 45 | -75.517 | 39.159 | 666168 | 61500 |
| Bismarck | ND | 46 | -100.767 | 46.805 | 638800 | 66100 |
| Washington | DC | 47 | -77.016 | 38.905 | 606900 | 138500 |
| Montpelier | VT | 48 | -72.572 | 44.266 | 562758 | 72600 |
| Cheyenne | WY | 49 | -104.792 | 41.145 | 453588 | 99300 |
